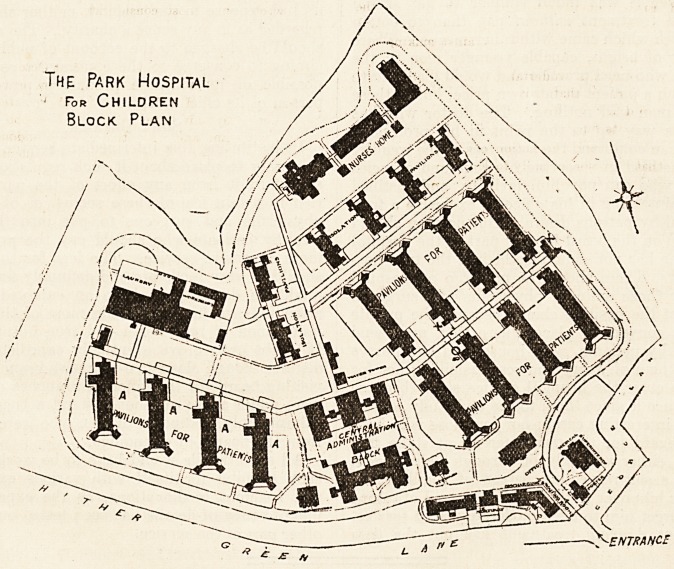# The Park Hospital for Sick Children

**Published:** 1910-12-03

**Authors:** 


### THE PARK HOSPITAL FOR SICK CHILDREN.

On Saturday, November 19, 1910, the Right Hon. J. Burns, President of the Local Government Board, opened the Park Hospital at Hither Green, S.E., as a hospital for sick children. This hospital was fully described in our issue of November 27, 1897, pp. 158-9, but as some modifications have been made since then it is worth while drawing attention to its salient features. From the interesting "Order of Proceedings" issued for the occasion by the Metropolitan Asylums Board we take the following particulars.

The hospital was built in 1895-97, the contractors being Messrs. Leslie and Co., Ltd., from plans by Mr. E. T. Hall, F.R.I.B.A., F.S.I., which had been selected from a number of designs sent in for adjudication in public competition. On July 12, 1897, the late King, then Prince of Wales, opened the institution. At that time the accommodation provided was for 548 fever patients and for a staff numbering 300 persons, and the total expense of building and equipment came to £280,000, which includes the expenses of renovation up to 1909.

When in July last the managers put forward for the consideration of the Local Government Board proposals for the utilisation of some of the vacant accommodation which was available in consequence of the considerable decline in the number of fever cases treated at the hospital, these proposals were favourably entertained. It appeared that there were no fewer than 2,049 children in the Metropolitan workhouses and infirmaries, of whom 837 were non-infectious and 561 infectious cases, which could be transferred to institutions under the Asylums Board if such were available. Accordingly the Board decided to allocate a hospital hitherto used as a fever hospital for these cases, and the Park Hospital was selected as the one which, besides being quite suitable for the purpose, could also from its geographical position be best spared from the infectious-hospital service. The hospital was completely disinfected, renovated, and reopened for the admission of sick children on the date above mentioned.

The Park Hospital occupies a high elevation on a site of about twenty acres at Hither Green. It is three-quarters of a mile distant from Lewisham and six miles from London Bridge, and within easy reach of the Hither Green station on the South Eastern line. It is built on the pavilion plan, the various buildings being arranged in a radial manner, and the pavilions themselves being connected by covered ways with the kitchen, stewards' department, water-tower, dispensary, and telephone exchange, which are located in the centre. The pavilions are two-storeyed, with fire- and sound-proof floors, and with their long axes running nearly wards. In each stack (which is externally made of glazed faience) are eight of these air-flues and four smoke-flues, and the whole are contained in an area 3 feet 6 inches square. All these air-flues can be swept, and all have outlets on two sides. The staff accommodation includes a nurses' home on the summit of the site, divided into three houses connected with corridors on every floor, a female servants' home containing a separate cubicle for each maid (central administrative block on plan), and a male-staff block on the opposite side of the quadrangle. Near by is the house for the assistant medical officers and a residence for the steward. To the south-east are placed the laundry, boiler and engine houses, workshops, disinfecting-house, well, and water-softening apparatus. The medical superintendent's house is near the entrance, and the offices, discharge-rooms, mortuary, etc., are close by. The hospital is supplied with water taken from the chalk, the water being pumped into a south and north. A feature of the design is that there is no internal communication between the two storeys, the upper one being reached by an external staircase opening from the covered ways. The larger buildings contain three wards on each floor, one for thirty patients, one for two beds, and one for one bed, together with the ward scullery, bathroom, and other offices. All the wards are heated by hot-water radiators in cases designed to admit fresh air, which air is then warmed and passed into the ward at a low velocity calculated to change the air in the ward three times per hour; the inlet of cold air can be adjusted to suit varying wind-pressure. In addition there are central open fireplaces and stacks of flues especially made for this hospital. In these central stacks every smoke-flue can be swept from the external basement. The smoke-flues are surrounded by aspirating-flues, which, being thus heated, induce an up-current, and so draw off the heated vitiated air from the centre of the softening apparatus alongside and then to the water-tower, which forms a central feature, and contains a four-dial clock visible from all parts of the premises. The hospital is lit by electricity generated on the premises.

During its thirteen years' use for fever-cases the Park Hospital has done excellent work for the Metropolis. No fewer than 24,248 cases of scarlet fever, 8,775 cases of diphtheria, 714 cases of enteric fever, and 3,854 cases of other diseases have been treated in the hospital, the first fever patient having been admitted on November 8, 1897, and the last patient discharged on September 2, 1910.

The sub-committee in charge of the hospital consists of Mr. R. Woolley Walden, J.P. (Chairman of the Children's Committee), Miss I. M. Baker (Vice-Chairman of the Children's Committee), Mr. W. G. Bevan (Chairman of the sub-committee), Dr. Elliott Browne, the Rev. F. H. Higley, and Messrs. W. E. Hinton and Thomas Bates. The Medical Superintendent is Dr. R. A. Birdwood, the Matron Miss S. A. Villiers, and the Steward Mr. H. Harrington.

## Figures and Tables

**Figure f1:**